# *NUP214* fusion genes in acute leukemia (Review)

**DOI:** 10.3892/ol.2014.2263

**Published:** 2014-06-18

**Authors:** MIN-HANG ZHOU, QING-MING YANG

**Affiliations:** Department of Hematology and Oncology, The First Affiliated Hospital of the People’s Liberation Army General Hospital, Beijing 100048, P.R. China

**Keywords:** *NUP214*, acute lymphoblastic leukemia, fusion gene, acute myeloid leukemia

## Abstract

Nucleoporin 214 (*NUP214)*, previously termed *CAN,* is required for cell cycle and nucleocytoplasmic transport. The genetic features and clinical implications of five *NUP214*-associated fusion genes are described in this review. *SET-NUP214* was most frequently observed in T-cell acute lymphoblastic leukemia (T-ALL), concomitant with the elevated expression of *HOXA* cluster genes. Furthermore, the fusion transcript may be regarded as a potential minimal residual disease marker for *SET-NUP214*-positive patients. Episomal amplifications of *NUP214-ABL1* are specific to T-ALL patients. The *NUP214-ABL1* gene is observed in ~6% of T-ALL, in children and adults. Targeted tyrosine kinase inhibitors plus standard chemotherapy appear to present a promising treatment strategy. *DEK-NUP214* is formed by the fusion of exon 2 of *DEK* and exon 6 of *NUP214*. Achieving molecular negativity of *DEK-NUP214* is of great importance for individual management. *SQSTM1-NUP214* and *NUP214-XKR3* were only identified in one T-ALL patient and one cell line, respectively. The *NUP214* fusions have significant diagnostic and therapeutic implications for leukemia patients. Additional *NUP214*-associated fusions require identification in future studies.

## 1. Introduction

Nucleoporin 214 (*NUP214)*, also known as *CAN*, is an FG-repeat-containing nucleoporin. The encoded protein is found on the cytoplasmic side of the nuclear pore complex, and is necessary for the cell cycle and for transport of material between the nucleus and cytoplasm (1). The *NUP214* gene is located at band 9q34.1 and includes 36 exons numbered 1–36. Several novel *NUP214* partner genes have been described recently, and the present study provides a review on this topic.

## 2. *SET-NUP214* fusion gene

The *SET* gene was previously termed *TAF-I* or *TAF-Iβ*. The encoded protein inhibits cell apoptosis caused by cytotoxic T lymphocytes (2). Del(9)(q34.11q34.13) (3), or occasionally t(9; 9)(q34; q34), leads to the formation of the *SET-NUP214* fusion gene, and often predicts a poor outcome for patients (4,5). The fusion gene is most frequently observed in T-cell acute lymphoblastic leukemia (T-ALL) (4,6), but rarely in acute myeloid leukemia (AML) (7) or acute undifferentiated leukemia (8). Similar to the *PICALM-MLLT10* fusion gene, *MLL* rearrangements and the inv(7)(p15q34) aberration (9–11), the *SET-NUP214* fusion gene contributes to the occurrence of T-ALL by increasing the expression of *HOXA* cluster genes (6). Two cell lines, the T-ALL LOUCY cell line and the AML MEGAL cell line, are known to exhibit the *SET-NUP214* gene (3). The *SET-NUP214* gene in cell lines is formed as a result of the fusion of exon 7 of *SET* and exon 18 of *NUP214*. In addition, the fusion of *SET* exon 7 and *NUP214* exon 17 has also been identified in leukemia patients. The fusion gene inhibits hematopoietic cell differentiation (12,13). However, concurrent chromosomal abnormalities are also required to induce the development of leukemia (4,14).

In a study of 256 ALL patients, two T-ALL patients with the *SET-NUP214* gene were identified using multiplex reverse transcription polymerase chain reaction (RT-PCR). Overexpression of the *HOX* genes (*HOXA7*, *HOXA9* and *HOXA10*) was also detected in the two patients (15). Wang *et al* (16) identified three patients with the *SET-NUP214* gene out of a total of 46 T-ALL patients. Notably, all three patients exhibited a mutation in *PHF*, a key tumor suppressor gene in T-ALL. An additional three patients with the *SET-NUP214* gene in a study by Van Vlierberghe *et al* (6) were found to exhibit the *NOTCH1* mutation, which occurs in almost 50% of T-ALL patients (17). Gorello *et al* (4) identified seven patients with the *SET-NUP214* gene in 152 T-ALL patients. All seven patients exhibited ≥1 additional genetic abnormality, and the majority of patients succumbed to the disease within two years of diagnosis. A significant correlation between minimal residual disease (MRD), detected by the *SET-NUP214* fusion transcript, and the clonal *Ig*/*TCR* rearrangements was identified in fifteen follow-up bone marrow samples obtained from three pediatric patients with the *SET-NUP214* gene (18). The consistency of the two methods showed that the *SET-NUP214* fusion transcript may be regarded as a potential MRD marker for *SET-NUP214*-positive patients.

## 3. *NUP214-ABL1* fusion gene

The *ABL1* gene is fused to the *BCR* gene in >95% of chronic myeloid leukemia (CML) patients (19). With the exception of the *BCR-ABL1* gene, the *NUP214-ABL1* gene is the most common fusion gene in hematological malignances involving the *ABL1* gene (20). The NUP214-ABL1 protein cannot activate the ABL1 kinase unless it interacts and competes with other nuclear pore proteins and thus, the amplification of *NUP214-ABL1* is necessary for neoplastic transformation (21). The episome is an extrachromosomal genetic element that has the ability to exist autonomously and freely replicate in the cytoplasm or be integrated with the chromosome and replicate with it (22,23). Episomal amplification of *NUP214-ABL1* is often evident in leukemia cells and varies even in the same patient, with 5–50 copies/cell (24,25). Episomes exhibiting the *NUP214-ABL1* gene are visible by fluorescence *in situ* hybridization (FISH) with specific probes or molecular analysis, but are undetectable by conventional cytogenetics (24).

The *NUP214-ABL1* gene is observed in ~6% of T-ALL, in children and adults (24). Patients with the *NUP214-ABL1* gene usually present with high-risk factors of T-ALL, including an elevated white blood cell count, a mediastinal mass and extramedullary involvement, often with early relapse and a poor outcome. The *NUP214-ABL1* gene is highly specific for T-ALL (21). The *NUP214-ABL1* gene has also been identified in B-cell ALL patients (26). Different types of the *NUP214-ABL1* gene have been found in patients with T-ALL. The most common gene found in previous studies was exon 31 of *NUP214* fused to exon 2 of *ABL1*, followed by exon 29 of *NUP214* fused to exon 2 of *ABL1*. The breakpoints of *NUP214* were variable, located between exon 23 and 34 (27–30). The *NUP214* gene was most frequently fused to exon 2 of *ABL1*, but rarely to exon 3 of *ABL1*. In addition, the fusion gene was observed in four cell lines (31), ALL-SIL and TALL-1024 (exon 32 of *NUP214* fused to exon 2 of *ABL1*) and PEER and BE-13 (exon 34 of *NUP214* fused to exon 2 of *ABL1*). The fusion gene was revealed by FISH at chromosome 9q34 as homogeneously staining regions and was found to replicate with the chromosome in all four cell lines. The fusion protein retains two coiled-coil domains of NUP214 and the tyrosine kinase domain of ABL1.

The development of acute leukemia with the *NUP214-ABL1* gene is partly due to the increased tyrosine kinase activity. Therefore, targeted therapy with specific tyrosine kinase inhibitors may be effective in the treatment of the disease (30,32). Imatinib, the first tyrosine kinase inhibitor, has considerable efficacy against CML exhibiting the *BCR-ABL1* gene (33). The *NUP214-ABL1* fusion is a late event and not the only aberration in T-ALL, often in combination with the deletion of the important tumor suppressor genes *CDKN2A* and *PTPN2* (34) and the overexpression of *TLX1* or *TLX3* (27,32), increasing the risk of a poor survival time (28). Therefore, in contrast to CML, monotherapy with imatinib is inadequate for treating T-ALL patients with the *NUP214-ABL1* gene. In addition, the easy and usual amplifications of the *NUP214-ABL1* gene on episomes are beneficial for the development of relapse and resistance. In a study by Clarke *et al*, a total daily dose of 600 mg imatinib was administered in combination with vincristine and prednisolone to a male T-ALL patient with the *NUP214-ABL1* fusion gene who relapsed three months after a sibling allograft (35). The patient achieved rapid hematological remission and remained in remission for six months prior to a secondary relapse. Overall, the patient exhibited a brief, but initially favorable response to imatinib. De Keersmaecker *et al* (36) revealed that the SRC family kinase LCK was crucial for the proliferation and survival of T-ALL cells with the *NUP214-ABL1* gene. Dasatinib and bosutinib, dual ABL1/SRC kinase inhibitors (37), are considered to be important in the treatment of *NUP214-ABL1*-positive disease. Deenik *et al* (38) reported the case of a young male T-ALL patient with the *NUP214-ABL1* fusion gene who was treated with dasatinib monotherapy (70 mg twice daily), while chemotherapy was postponed due to the surgical removal of a ruptured spleen. The patient achieved a complete hematological response and cytogenetic remission three weeks later. Therefore, dasatinib in combination with standard chemotherapy appears to present a promising treatment strategy.

## 4. *DEK-NUP214* fusion gene

*DEK* is involved in DNA duplication and mRNA processing. The *DEK-NUP214* gene, which results from t(6;9)(6p22.3;9q34.1), is associated with 1% of AML and myelodysplastic syndromes (39,40). Sandén *et al* (41) demonstrated that the *DEK-NUP214* gene increased cell proliferation via the upregulation of mammalian target of rapamycin complex 1 (mTORC1) activity, and that the *DEK-NUP214* induced proliferation was reversed by the mTORC1 inhibitor. Therefore, the mTOR inhibitor may be suitable for the treatment of the patients with the *DEK-NUP214* gene. The *DEK-NUP214* gene is generated from the rare fusion between exon 2 of *DEK* and exon 6 of *NUP214* (42). Patients with this fusion gene are characterized by a young age, marrow basophilia, preceding myelodysplasia and a poor prognosis (39,43,44). It has been found that ~70% of patients with the fusion gene exhibit internal tandem duplications of the tyrosine kinase *FLT3*, as well as higher numbers of white blood cells and bone marrow blasts, and markedly lower complete remission rates (39,45). The *DEK-NUP214* gene is most frequently observed in patients with AML-M2, according to the French-American-British classification (44).

Garçon *et al* (46) applied the quantitative PCR (qPCR) method to analyze 79 bone marrow and peripheral blood samples of 12 patients (ten AML and two myelodysplastic syndrome patients) with the *DEK-NUP214* gene. Five patients exhibited an absence of the *DEK-NUP214* gene (sensitivity, <10^5^). All five patients underwent allogeneic hematopoietic stem cell transplantation (allo-HSCT) and four showed consistent molecular negativity with a median follow-up time of 18.5 months. By contrast, the additional seven patients who did not achieve *DEK-NUP214* negativity all succumbed to the disease following a median time of 12 months from diagnosis. It was demonstrated that monitoring the *DEK-NUP214* fusion transcript by qPCR was a useful method for individual management. Four patients with the positive *DEK-NUP214* gene had survived prior to transplantation, indicating that allo-HSCT may overcome the poor prognosis of the *DEK-NUP214* fusion gene and that allo-HSCT is critical for the increased survival times of patients with the *DEK-NUP214* gene.

## 5. *SQSTM1-NUP214* fusion gene

The protein encoded by *SQSTM1* mediates the activation of the nuclear factor-κB signaling pathway in response to upstream signals (47). Gorello *et al* (48) reported the case of a 20-year-old male with chemoresistant T-ALL, with an overall survival time of 16 months. Gene expression profiles showed that the patient was clustered tightly with the *SET-NUP214*-positive T-ALL patients, exhibiting an elevated expression level of the *HOXA* cluster genes (*HOXA7*, *HOXA9* and *HOXA10*). However, the patient exhibited certain common clinical characteristics with the *SET-NUP214*-positive patients, including an immature phenotype and a poor outcome (4). Metaphase FISH revealed an unbalanced translocation, der(5)t(5;9)(q35;q34). Furthermore, RT-PCR and sequencing confirmed a novel fusion gene with exon 5 of *SQSTM1* fused to exon 33 of *NUP214*. In contrast to the *SET-NUP214* gene with 42/44 NUP214 FG repeats (49), the *SQSTM1-NUP214* gene exhibited only 14/44 FG repeats (50) and thus, the leukemogenic mechanisms of the two *NUP214* fusion genes appeared to be markedly different. A total of 136 T-ALL patients were screened by nested RT-PCR, and no other patients with the *SQSTM1-NUP214* gene were identified, suggesting that the fusion gene was an extremely rare event in the T-ALL patients. Further study on the incidence and clinical implications of the *SQSTM1-NUP214* gene in ALL is required.

## 6. *NUP214-XKR3* fusion gene

*XKR3* is a membrane transporter in the XK/Kell complex of the Kell blood group system, located at chromosome 22q11.1 (51). Levin *et al* (52) investigated gene fusions in the cDNA Illumina data (Illumina, Inc., San Diego, CA, USA) of K562 (a CML cell line) using targeted RNA sequencing. In addition to the *BCR-ABL1* fusion gene, a novel *NUP214-XKR3* fusion gene was identified in the cDNA library. A total of four *NUP214-XKR3* fusion transcript isoforms were detected, and all four transcripts were confirmed by Sanger sequencing RT-PCR. However, only the fusion gene between exon 29 of *NUP214* and exon 4 of *XKR3* retained an open reading frame downstream of the fusion gene. However, the functional significance of the fusion gene was not reported in the literature and the occurrence of the *NUP214-XKR3* gene in leukemia patients has not yet been reported.

## 7. Conclusion

In the present review, five *NUP214*-associated fusion genes that have been identified in leukemia patients were described. The majority of the fusion genes were observed in T-ALL patients. Identifying *NUP214* fusions is extremely important due to the diagnostic and therapeutic significance for leukemia patients. The *SQSTM1-NUP214* and *NUP214-XKR3* fusion genes were described in only one patient and one cell line, respectively. To investigate the incidence and the clinical implications in leukemia patients, further investigations are required. Additional partner genes of *NUP214* remain to be identified in the future.
